# Extracellular Vesicles in Regeneration and Rehabilitation Recovery after Stroke

**DOI:** 10.3390/biology10090843

**Published:** 2021-08-30

**Authors:** Alice Gualerzi, Silvia Picciolini, Francesca Rodà, Marzia Bedoni

**Affiliations:** IRCCS Fondazione Don Carlo Gnocchi Onlus, 20148 Milan, Italy; spicciolini@dongnocchi.it (S.P.); froda@dongnocchi.it (F.R.); mbedoni@dongnocchi.it (M.B.)

**Keywords:** stroke, extracellular vesicles, rehabilitation, regeneration, precision medicine, biomarkers, recovery

## Abstract

**Simple Summary:**

Patients that survive after a stroke event may present disabilities that can persist for a long time or permanently after it. Clinical intervention with pharmacological and rehabilitation therapies must follow the correct timing and dosing to guarantee optimal recovery of the patients. Extracellular vesicles are nanometric cell products that can be detected in body fluids such as blood and urine; their use as biomarkers for the personalization of stroke therapy and rehabilitation (rehabilomics) might help clinicians and patients to reach the optimal recovery and ameliorate patient quality of life. Moreover, extracellular vesicles released by cells such as stem cells or other human cells are under investigation for their possible regenerative role that could be coupled to standard therapies to stimulate brain remodeling and ameliorate the recovery after stroke. In this review we describe some of the most recent advancements in the field and discuss the potentialities of extracellular vesicles in brain regeneration and rehabilitation after stroke.

**Abstract:**

Patients that survive after a stroke event may present disabilities that can persist for a long time or permanently after it. If stroke prevention fails, the prompt and combinatorial intervention with pharmacological and rehabilitation therapy is pivotal for the optimal recovery of patients and the reduction of disabilities. In the present review, we summarize some key features of the complex events that occur in the brain during and after the stroke event, with a special focus on extracellular vesicles (EVs) and their role as both carriers of biomarkers and potential therapeutics. EVs have already demonstrated their ability to be used for diagnostic purposes for multiple brain disorders and could represent valuable tools to track the regenerative and inflammatory processes occurring in the injured brain after stroke. Last, but not least, the use of artificial or stem cell-derived EVs were proved to be effective in stimulating brain remodeling and ameliorating recovery after stroke. Still, effective biomarkers of recovery are needed to design robust trials for the validation of innovative therapeutic strategies, such as regenerative rehabilitation approaches.

## 1. Introduction

The World Health Organization (WHO) defines stroke as a clinical syndrome characterized by a rapid onset of focal cerebral deficit lasting more than 24 h or leading to death [1]. It is the second cause of death according to the WHO Global Health Estimates and the fourth cause of disability for over 65-year-old people [2]. Patients that survive after a stroke event may present disabilities that can persist for a long time or permanently after it, negatively affecting the quality of life of stroke patients. The most common are motor impairment (hemiparesis, hemiplegia), central facial paresis, language and speech disorders, and global and mixed aphasia and dysarthria. Less commons disorders include altered levels of consciousness, impaired vision, and decreased blood flow to some parts of the brain [3].

To prevent stroke, the identification of the risk factors for stroke is essential. The current WHO guidelines advocate a combination of population-wide and high-risk approaches. The first one targets behavioral and lifestyle risk factors (e.g., tobacco smoking, diet, and nutrition) that can positively influence the risk for stroke and many other diseases. Hypertension and diabetes mellitus are two leading risk factors for stroke, and the latter is a predictor of worse long-term outcomes. Additionally, overweight and obesity, hyperhomocisteinemia, aging, and some genetic factors can increase the risk of stroke outbreak, while it is diminished by routine physical activity thanks to exercise-induced increase in the expression of neuroprotective factors such as endothelial nitric oxide synthetase, brain-derived neurotrophic factor (BDNF), and insulin-like growth factor 1 (IGF-1) [2]. Concomitantly, surveillance and screening are important activities to prevent stroke injuries. In particular, the identification of high-risk stroke patients and their education for decreasing the risk of an acute stroke event is performed through lifestyle evaluation, physical examinations, and laboratory measurements, also taking into account gender differences; it has been shown that males are more predisposed to having a stroke, while females have a higher fatality risk. In fact, females live longer (aging increases the probability to have stroke), have higher blood pressure due to pregnancy or birth control medications, and they are more subjected to depression and anxiety with psychosocial factors being involved in stroke outbreak. If the subject is stroke-prone, primary prevention can be done including monitoring blood pressure, maintaining a healthy lifestyle (physical exercise, healthy diet, no smoking, and no alcohol abuse), and taking medications for hypertension, high cholesterol, or atrial fibrillation (antiplatelets, anticoagulants) if necessary [2]. 

Early and precise diagnosis is required to accurately select the optimal intervention in acute stroke patients. At the same time, to reduce the risk for impaired motor and cognitive functions, the enhancement of nervous system recovery is crucial to help stroke patients to go back to their previous life. This is achieved through the combination of pharmacological and rehabilitation strategies that favor and sustain spontaneous regenerative processes occurring after injury. In recent years, a new term was created to identify a new discipline that combines physical rehabilitation and regenerative medicine, i.e., **regenerative rehabilitation**. This is meant to describe a branch of medicine that is aimed at the restoration or establishment of normal function after loss due to injury, such as stroke events, aging, or congenital defects [4,5]. Regenerative rehabilitation pairs exercise principles with regenerative therapies to facilitate and enhance regeneration and repair, but only a few studies have recently reported about the combination of cell therapy and motor rehabilitation to enhance recovery [6].

Independently from the treatment, recovery after stroke is difficult to predict, and the search for specific predictive biomarkers for the design of optimal and personalized therapeutic strategies is still in its infancy.

In this review, we will firstly summarize some key features of the complex events that occur in the brain during and after the stroke event, we will introduce extracellular vesicles (EVs) as emerging carriers of brain biomarkers, and then we will focus on the EV potentialities as facilitators and biomarkers of stroke recovery. 

## 2. Stroke Induced Response to Injury 

There are two main types of stroke with different etiopathology and management, namely the hemorrhagic stroke (HS) and the ischemic stroke (IS). HS is caused by the rupture of a blood vessel inside the brain, and it can be divided into intracerebral or subarachnoid types [2]. The intracerebral HS has the highest mortality rate of all stroke types, and it can be sub-classified into two major subtypes (primary or secondary) according to the cause of bleeding [7]. On the other hand, the subarachnoid HS occurs spontaneously after the rupture of a cerebral aneurysm or head injury [2,3]. IS is the most common type, representing 85% of all stroke cases. Unlike HS, it is characterized by the blockage of a brain’s artery due to the aggregation of platelets, forming fibrin meshes [3]. The main causes of IS are intracranial thrombosis, extracranial embolism, the rupture of a plaque in an atherosclerotic disease, a dislodged embolism formed in the heart, and the occlusion of small penetrating cerebral arteries due to cerebral small vessel disease. The occlusion of the vessel results in a hypo-perfused area of the brain tissue and a surrounding but salvageable area called ischemic penumbra. Cerebral blood flow disorder duration and time of occlusion determine the degree of the neurological dysfunction [8]. Both types of stroke cause local hypoxia that damages the brain tissue, with varying severity depending on the site and duration of the hypoxic state. 

**Figure 1:**
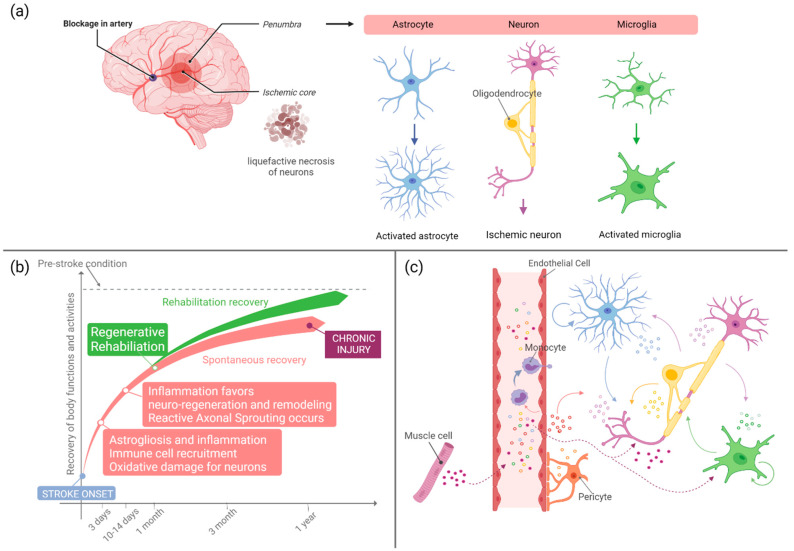
(**a**) Schematic representation of the cellular events occurring in the acute phase that follows a stroke event. Neurons, astrocytes, and microglia are the neural cells mostly involved in the response to injury. Astrocytes and microglia modify their metabolism and morphology towards an activated phenotype. Neurons in the ischemic core might undergo irreversible damage and liquefactive necrosis, while in the penumbra, salvageable neurons undergo acidophilic transformation and oxidative damage. (**b**) Concise summary of some of the main events occurring after stroke. Regenerative rehabilitation can significantly ameliorate stroke patient conditions leading to better therapeutic outcome and functional recovery. (**c**) Schematic illustration of the EV-mediated crosstalk occurring between neural and endothelial cells after stroke. Monocytes are recruited in the injured area. Rehabilitation and exercise stimulate EV release by skeletal muscle cells; muscle EVs that reach the brain tissue can favor recovery and resolution of inflammation. Created with BioRender.com.

From a biochemical point of view, stroke causes damages in different cell types within the nervous tissue, inducing morphological and signaling changes (Figure 1a). Neurons are the most susceptible cells to the decrease in blood flow and the consequent lack of glucose and oxygen supplementation, as their metabolism almost exclusively depends on oxidative phosphorylation. While neurons in the ischemic region undergo liquefactive necrosis where axons and bodies disappear, those in the penumbra are called ischemic neurons or red neurons, and they are characterized by acidophilic cytoplasm, transformation of neuronal proteins, disruption of endoplasmic ribosomes, and Nissl bodies. Furthermore, the absence of ATP production causes the dysfunction of ATP-dependent ion transport pumps, leading to the depolarization of neurons and intracellular increase of sodium, chloride, and calcium ions and a decrease of potassium. This leads to mitochondrial damage, disruption of the plasma membrane, DNA fragmentation, and the overproduction of radicals, bringing oxidative stress. Neurons are the most vulnerable cells to radicals, as they have low antioxidant enzyme activity, high concentrations of peroxidable lipids, high oxygen consumption, and high iron levels. Moreover, the excessive release of glutamate (an excitatory neurotransmitter) and the inhibition of its re-uptake cause high glutamate levels in the synaptic and pre-synaptic sites that lead to neuron death through a phenomenon called excitotoxicity. Lastly, the influx of water inside neurons leads to their swelling, causing so-called cytotoxic edema. The degree of neuron damage depends on their location, as neurons in grey matter are more susceptible to injury than neurons in white matter [1,3]. In a temporal perspective, soon after the insult, the above described processes induce cell fragmentation and dissolution, and inflammation is triggered involving both astrocytes and endothelial cells. In normal conditions, astrocytes contribute to the regulation of cerebral blood flow by their direct connection to blood vessels, supply energy metabolites to neurons, participate in the synaptic function, and regulate ion and fluid balances. After three days from the stroke event, astrocytes undergo a reactive gliosis also called astrogliosis where they temporarily or permanently change their gene expression and morphology. They start exhibiting glial fibrillary acidic protein (GFAP) and other factors; on one hand, astrocytes help ischemic recovery by decreasing inflammation and supporting neuronal recovery in the penumbra, while on the other hand, astrogliosis increases damage in the central nervous system [1,3]. Concomitantly, microglial cells start releasing inflammatory proteins such as cytokines, chemokines, proteases, cyclooxygenase 2, reactive oxygen species, prostaglandins, and other metabolites that lead to the increase of circulating monocytes. Microglial cells are activated within a few minutes of stroke outbreak, but their role is not completely understood yet. Actually, different types of microglial cells are known to release different factors, which can be neuroprotective or neurotoxic, making the role of microglia both dual and controversial in stroke as in many other brain disorders [9]. 

Until about 10–14 days after stroke, the induced inflammation has a beneficial role as it helps the creation of an extracellular milieu that favors subsequent axonal entry and re-innervation [10] (Figure 1b). At the same time, inflammation can bring cerebral edema that can result from vasodilation and increased blood brain barrier (BBB) permeability (vasogenic edema) or from swelling and death of cells due to a severe ionic load and inadequate metabolism (cytotoxic edema) [10]. For these reasons, the role of inflammation in brain tissue regeneration is somewhat controversial, being that some components of inflammation are harmful for neurons while others can promote cell survival, axon sprouting, and regeneration. 

Staring from 2 weeks after the stroke event, neurogenesis is stimulated, and immature neurons are recruited into damaged areas. Such recruitment and regeneration processes can last up to several months after injury, maintaining the neural environment conductive to recovery thanks to enhanced neurotrophic factors, loose extracellular space, etc. After 6 months to 1 year, stroke injury may be considered chronic, with reduced opportunities for remodeling and regeneration (Figure 1b).

Prompt intervention with pharmacological and rehabilitation treatment is crucial to stop the negative escalation of events that follow a stroke event and to favor a positive niche for the regeneration of the damaged tissue and the restoration of physiological functions.

## 3. Current Stroke Diagnosis, Profiling, and Therapeutic Strategies

As stroke is a very complex entity, its identification and diagnosis pass through different phases. Firstly, patient history and physical conditions are examined. Then, if they suggest stroke injury, the patient is subjected to a neurological examination. The diagnosis of stroke and its proper classification has two aims: to distinguish stroke from stroke mimicking diseases (such as brain tumors, metabolic, infectious or psychological disorders), and to discriminate ischemic from hemorrhagic stroke, as they require different treatments. Currently, diagnostic tests based on brain imaging, i.e., computed tomography (CT) and magnetic resonance imaging (MRI) are the only techniques that can distinguish between HS and IS, evaluating the extent of the brain lesion, even though CT scanning is not sensitive to old hemorrhages, which require MRI and digital subtraction angiography [2]. Some point-of-care devices, such as an on-board CT scanner, might enable prehospital diagnosis in order to initiate the treatment as early as possible. Indeed, time of intervention is fundamental for patients that are in the acute stroke phase, where the aim of treatments is to restore blood flow as soon as possible in order to save brain tissue from severe injury. It has been estimated that in IS, each minute saved between symptom onset and treatment initiation saves 1.9 million neurons and grants about 4 days of extra healthy life [8]. Clinical scales such as the National Institute of Health Stroke Scale (NIHSS) for the quantification of stroke severity are used to identify the severity of stroke but fail in the distinction between HS and IS. Another way to improve the evaluation of the pathophysiological state of stroke patients could be represented by clinical biomarkers. A good biomarker candidate for this purpose should be sensitive to IS and specific to the brain. Potential biomarkers for ischemic stroke, according to its characteristic pathophysiology, are those released by activated astrocytes, damaged neurons, systemic inflammatory responses, and a dysfunctional endothelium. The BBB breakdown that occurs after stroke allows the release of proteins from astrocytes and neurons into the bloodstream and the possibility to be used as disease biomarkers. In recent years, many studies investigated the possibility to use inflammatory markers, cytokines, microRNA, and other small molecules in the diagnosis, prognosis, and treatment monitoring of stroke patients; however, the controversial clinical findings on these biomarkers have not allowed their entry in clinical practice yet [11]. 

In the acute and subacute phase, most drugs are aimed at normalizing the electrochemical balance of the brain in order to minimize secondary injury. Additionally, suppression of glutamatergic activity appears to be beneficial as well as the modulation of GABAergic inhibition [10]. Blood pressure lowering therapies can improve the outcome of HS patients, while they would be harmful for IS patients, for whom reperfusion therapies are used. Reperfusion treatments greatly improve patients’ outcomes if started within 4.5 to 6 h after the ischemic stroke onset. Unfortunately, most patients arrive too late at the hospital, and only on a minority of them reperfusion therapies are used. Treatment initiation with neuroprotective therapies during the pre-hospital phase would improve the functional outcome of patients [8]. 

Therapies for the acute phase can be a combination of preventive treatments and tissue plasminogen activator (tPA). tPA is the only FDA-approved therapy for IS, but it is highly time-dependent. Patients must take it within 4.5 h from the onset of symptoms, otherwise they undergo hemorrhagic transformation. Another possible treatment is thrombectomy to physically remove the clot. Therapies that are being developed include the use of mi-RNA to silence or activate genes or proteins in the post-stroke brain and stem cells for regenerative rehabilitation [2,3]. 

One third of stroke survivors presents recurrence within five years. To prevent a relapse, patients are suggested to follow a healthy lifestyle (that includes the same indications of the preventive phase), antiplatelet or lipid-lowering therapies, and, in specific cases, carotid stenosis surgery. Self-management and family support are important too [2]. Last but not least, the **rehabilitation phase** is fundamental, as stroke is an acute event that has long-term consequences, and half of the survivors remain disabled after stroke has occurred. Rehabilitation can be based on physical, occupational, speech, or recreational therapies [2] but must be tailored to the patients’ clinical features in order to achieve the best results. For this reason, after acute treatment, stroke patients undergo neurological examination and profiling using validated clinical scales that can measure different parameters. The most used clinical scales for stroke are the Visual Analog Scale (VAS) and the Numerical Rating Scale (NRS) that measure the intensity of pain [12], Blaylock Risk Assessment Screening that analyses the risk of longer hospitalization [13], the Barthel index that measures levels of functional independence [14], and the Cumulative Illness Rating Scale (CIRSS), which evaluates comorbidities of stroke patients [15]. Based on clinical profiling, the rehabilitation program is determined. In Italy, the Italian Society for Physical and Rehabilitative Medicine has validated a protocol for the minimal evaluation requirements for stroke patients before the rehabilitation treatment assessment [16]. Moreover, in the attempt to improve the quality of life of patients and enhance rehabilitation efficacy, also the treatment of depression is crucial to optimize recovery as symptoms of depression and learned helplessness can be found in up to 30% of stroke patients [10]. 

## 4. Recovery after Stroke

Recovery is, of course, a complex process that occurs thanks to a combination of both spontaneous and learning-dependent mechanisms. Recovery includes the restoration of the functionality of damaged neural tissue, the reorganization of neural pathways, and the improvement and compensation of disparity between impaired skills and environmental demands [17]. Stroke recovery is heterogeneous in its nature; size and site of the lesion influence the long term effect of stroke, but individual recovery patterns differ from one another, making a patient′s outcome difficult to predict.

Stroke triggers a remarkable degree of plasticity in structural and functional connections, with axonal sprouting occurring in the cortical areas adjacent or connected to the infarct and its role in functional recovery depending on the nature of the original stroke [18]. At present, there are no biomarkers for axonal sprouting, as no molecular marker of a “regenerating” axon could be found in experimental models of stroke, even though candidate regeneration-associated or axonal sprouting genes, such as GAP43, are often expressed in dendrites or in non-neuronal cells such as astrocytes and oligodendrocytes [18]. 

As thoroughly reviewed by Carmicheal and colleagues [18], the remodeling of brain tissue after stroke involves three different axonal sprouting mechanisms: (i) reactive axonal sprouting; (ii) reparative axonal sprouting; and (iii) unbounded axonal sprouting. 

The local axonal sprouting into brain tissue, directly adjacent to the infarct, is termed *reactive axonal sprouting* and is part of the tissue reorganization and scar formation of the stroke itself. Reactive axonal sprouting is a reparative mechanisms preserved across species as part of the endogenous brain tissue remodeling after stroke, i.e., an automatic cellular response to neuronal injury that may play a role in this spontaneous recovery process [18]. Conversely, *reparative axonal sprouting* is a longer distance axonal sprouting that can be stimulated by blocking glial growth or inducing a neuronal growth program and is clearly associated with behavioral recovery. The differences in the specific brain areas where these two processes occur indicate the existence of molecular or cellular pattern communication mechanisms that guide the sprouting response after stroke, which does not occur in random directions.

The limited capacity of the adult brain to form new connections after stroke can be increased by blocking glial growth inhibitors or stimulating a neuronal growth program, manipulating behavioral activity through constraint-induced movement patterns or intensive skilled reach training. These actions might lead to *unbounded axonal sprouting* after stroke, a process that can degrade behavioral recovery. This maladaptive sprouting was suggested to occur when intensive neurorehabilitation was started too close to the time of the stroke, suggesting that further studies are needed to guide axonal sprouting with the right factors (drugs or biologics) and the right timing into functionally meaningful circuits [18].

To enhance recovery, rehabilitation and pharmacological therapy work synergistically, but a key factor for stroke rehabilitation efficacy is the intensity of training, especially in the acute phase, to enhance functional restoration and prevent inactivity-related complications. However, duration, intensity, and frequency of rehabilitation programs can vary significantly because of the patient-specific clinical picture. For this reason, the positive outcome of the rehabilitation processes depends on the ability of the clinicians to discern the individual levels of impairment and responses to treatment with simple, robust, and effective methods. Timing of delivery of the axonal sprouting therapeutic and timing of neurorehabilitative therapy are essential for optimal functional recovery. Early (within the first week of stroke) and simultaneous delivery of increased behavioral activity and axonal sprouting therapeutic can cause unbounded axonal sprouting and behavioral deterioration; on the other hand, sequential administration of axonal sprouting therapeutic and then rehabilitative training can produce substantial functional recovery, but much research needs to be done to define the timing of these two approaches, and the timing might differ according to the specific mechanism of action of a candidate axonal sprouting therapy [18].

Motor rehabilitation aims at maximizing the recovery and independence in daily living by discouraging dysfunctional compensatory behaviors and promoting the re-learning of appropriate motor control strategies. For this, rehabilitation protocols must adapt to the individual dynamics of recovery [19]. Among the clinical scales used for stroke patients′ evaluation, the Fugl–Meyer is one of the most adopted measures of motor impairment after stroke. Still, the precision of clinical tests is limited by inter-rater and intra-rater reliability, and their application can be limited by the considerable amount of time to be administered. For these reasons, instrumental approaches (e.g., neuro-biomechanical assessment) have been proposed to be integrated with clinical scales assessments; nonetheless, they are often too specific in the investigation of a single domain of the neuro-musculoskeletal system providing detailed but sectorial assessment. Conversely, merging all the domains is desirable in order to provide a comprehensive framework for complete and quantitative patient profiling. The use of an instrumental assessment followed by a multimodal approach was used for the identification of quantitative neurophysiological metrics correlating with clinical measures (e.g., FM,A and grip force) [19]. The combined analysis of kinematic, muscular, and brain activity provided a good and accurate patient characterization in line with the outcome of the clinical scales. Indeed, a key impediment in the development of new therapeutic strategies is knowing the perfect timing for intervention and exact profiling of patients [20]. Objective biomarkers are needed as a measure of underlying molecular and cellular processes occurring after stroke and to predict recovery and/or treatment response. The evaluation of the stroke patient is crucial to assess the therapeutic strategy and the personalized rehabilitation program. It must be as complete as possible, including all comorbidities and habits that influence the health status, while still remaining translatable to multiple clinical settings and comprehensible for all specialists involved in the rehabilitation path.

**Figure 2:**
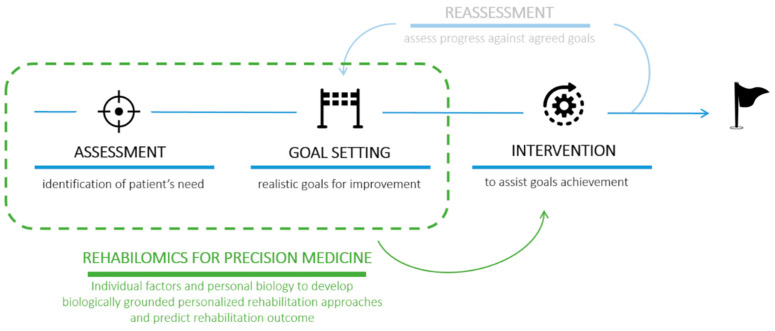
Schematic representation of the 4 phases of the rehabilitation process involving (**1**) assessment, (**2**) goal setting, (**3**) intervention, and (**4**) reassessment [17]. The introduction of the rehabilomics approach could accelerate the identification of a personalized intervention (precision medicine) in order to overcome the “reassessment” step (shaded).

As thoroughly described by Langhorne in 2011 [17], stroke rehabilitation is currently based on a cyclical process that involves four phases: (1) identification of patients’ needs ”assessment”, (2) goal setting to define the goals for the improvement of patients’ states that should be realistic and achievable, (3) intervention to assist patients in achieving the proposed goals, and (4) “reassessment”, redefinition of needs and goals based on progress and positive/negative outcomes of the intervention (Figure 2). It is now well-known that task-oriented training can assist the natural functional recovery of patients, supporting the idea that adaptive strategies compensate impaired body functions. What is more, training should be preferably conducted in the patient’s own context or environment, as good rehabilitation outcomes seems to be strongly associated with significant patient motivation and family engagement.

One key feature of a good rehabilitation is the multidisciplinary team care that involves medical, nursing, physiotherapy, speech therapy, and social work staff to assist patients and coordinate work, bringing better results in terms of patient survival, return to home, and regaining independence in daily activities [17]. The rehabilitation programs for stroke patients include multiple interventions for motor and cognitive impairments. The rehabilitation plan might be designed selecting specific treatments, such as bilateral training, constraint-induced movement therapy at modified doses, electrical stimulation, high-intensity therapy, repetitive task training, robotics, splinting, electromyographic biofeedback, electrical stimulation, mental practice, and repetitive task training, even though some of them raise concerns about their benefits to recovery [17]. For example, robot-assisted rehabilitation is emerging as a valuable tool for stroke patients’ recovery, but the effects of robotics alone or in conjunction with conventional therapy have not been thoroughly investigated yet. Although the use of robotic rehabilitation in addition to conventional therapy is currently recommended, a measurable biomarker that can objectively characterize the patient and monitor his/her recovery would favor prompt intervention, which is crucial to guarantee optimal recovery of the patient; comparison of rehabilitation and pharmacological protocols; and identification of personalized treatment to maximize recovery and ameliorate patients’ quality of life [16,21].

## 5. Extracellular Vesicles in Stroke

### 5.1. Extracellular Vesicle General Features and Clinical Application

EV is the generic term introduced by the International Society for Extracellular Vesicles (ISEV) to indicate particles naturally released from cells, delimited by a lipid bilayer, unable to replicate (as they do not have a functional nucleus), and found in different body fluids (such as blood, saliva, urine, cerebrospinal fluid, amniotic fluid, and breast milk) [22]. The classification of EVs can be based on physical characteristics, such as dimension (small if their size is between 100 and 200 nm and medium/large if they are bigger than 200 nm) and density (medium or high), biochemical composition, and cell of origin [22]. Based on their biogenesis, EVs can be also distinguished as exosomes, microvesicles, and apoptotic bodies [23]. Microvesicles have a size range between 100 and 800 nm and are shed by the plasma membrane of viable cells [24,25]. Apoptotic bodies have a size range between 200 nm and 5 µm and are shed from the plasma membrane of dying cells going through apoptosis [24]. The smallest EVs are called exosomes. They have a size range of 30–100 nm and are released by different types of cells (such as immune, tumor, or brain cells). They are pelleted with 100,000 g centrifugation and they have a density range between 1.10 and 1.20 g/cm^3^. They are formed by inward budding of compartments in the endocytic pathway, resulting in multivesicular bodies that fuse with the plasma membrane and release intraluminal vesicles, the exosomes [24]. EVs have a complex composition; they contain RNA, DNA, proteins, and lipids. All EVs share common characteristics even though in recent decades, further markers have been investigated, which are enriched in specific types of EVs, such as exosomes and microvesicles [22]. 

In recent years, EVs have gained more and more attention, as they represent the molecular signature of the cells from which they originated and can cross most anatomical barriers, being exploited to obtain information on barely accessible organs. For instance, EVs can be used very easily with a minimally invasive procedure, called a liquid biopsy, as biomarkers to access the brain state [26] and understand complex neurological conditions [27] as they can cross the BBB in both directions [26]. EVs can change their composition also according to the state of the cell of origin. Different studies demonstrated that EVs released by cells in modified cultures mimicking different physiological and pathological states have different biochemical compositions according to the environments in which cells are grown [28]. Further studies showed that EVs isolated from body fluids are carriers of molecules implicated in neurodegenerative, metabolic, and infectious diseases as well as cancer. In addition to their molecular composition, EV concentration can also provide information on the pathological state of the brain, as it was found to be increased during the inflammation process correlated with neurological diseases [27].

EVs also play an active role in intercellular communication by carrying soluble mediators such as cytokines [26] and being vectors of genetic information able to modify the range of genes in the recipient cells [28,29]. Thanks to their cell-to-cell communication role, in the central nervous system EVs were demonstrated to maintain physiological homeostasis; mediate cellular proliferation, differentiation, senescence, and synaptic activity; allow clearance of unwanted materials and cellular waste; and likely improve neuroprotection and regeneration in brain diseases [23,30,31]. 

In many neurodegenerative diseases, EVs play an important role in spreading misfolded proteins such as β-amyloid and Tau protein in Alzheimer’s disease, α-synuclein in Parkinson’s disease, and TDP-4s in amyotrophic lateral sclerosis [23,32]. Furthermore, in brain cancer advancement, tumor-derived EVs release soluble factors and improve the signaling that leads to cell growth dysregulation and development of hypoxic environments, which characterize cancer cells [23]. EVs have also been shown to play a part in immunomodulation, through the presentation of specific antigens to antibodies, such as those expressed by EVs derived from oligodendrocytes and endothelial cells in multiple sclerosis. 

It must be noted that in the EV context, compared to conventional biomarkers, the importance of pre-analytical variables is increased because of the difficulty of many techniques to work in their optimal sensitivity range, with many traditional instruments remaining limited in their capacity to discriminate EVs from instrument noise, and thus making artifacts exert major impact on all subsequent information. For this reasons, many position papers, guidelines, and road maps from the ISEV community [22,33,34,35] have been published, as have emerging techniques for the easy translation of EV-based biomarkers to the clinical setting [25,36,37]. 

Moreover, thanks to their intrinsic biocompatibility, biodistribution, and innate stability, EVs have been also investigated as drug delivery tools. EVs are potential therapeutic agents, exploiting their native biological functions, for example for regenerative medicine, cancer therapy, and immune modulation. For instance, EVs isolated from mesenchymal stem cells (MSC) can carry proteins, genetic material, growth factors, and lipids to a target cell, called a recipient cell, overcoming limits of safety that characterize cell transplantations [25] (see below). Several authors have investigated also the possibility to modify the EV cargo, loading specific therapeutic molecules within EVs for regenerative medicine applications, but technical limitations related to the nanometric size of vesicles have hampered our understanding of the molecular mechanisms underlying the packaging of cargo within EVs, their targeting towards specific disease sites, and their uptake [38]. 

### 5.2. Extracellular Vesicles in Stroke Pathophysiology

In recent years, the role of EVs in cell-to-cell communication within the brain tissue has emerged, with remarkable discoveries related to their involvement in neurodegenerative diseases [39,40,41] and microglia communication [9,42,43]. In parallel with such relevant studies, some authors started investigating the EV role in the inflammatory, regenerative, and reparative mechanisms activated after stroke injury (Figure 1c). 

The proteomic and miRNA analyses of EVs released by cultured primary cerebral endothelial cells and neural progenitor cells harvested from non-ischemic and ischemic animals revealed that stroke changed the EV cargo. In particular, EVs from ischemic animals were proved to mediate the stimulation of endothelial cells, favoring migration and capillary tube formation, and of neural progenitor cells, stimulating proliferation and neuronal differentiation, with synergic activity of endothelial and neural cells on neurogenesis and angiogenesis during brain repair [44]. After IS, neurons send specific “help me” signals by EV release in order to control microglial activation and influence microglial function. In particular, miR-98-loaded EVs were proposed to influence microglia phagocytic activity, preventing the destruction of stressed but salvageable neurons [45]. The expression level of miR-98 in EVs was found to be high on the first day after stroke but dropped significantly on the third day post-ischemia, suggesting that miR-98 could serve as an endogenous protective factor for the acute phase of IS [45]. 

Moreover, by the analysis of EVs released into the circulation after stroke, an increase of total EV levels (large and small vesicles) was observed. Those blood EVs were proved to contain pro-inflammatory proteins co-expressed with canonical EV markers, suggesting an altered inflammatory profile of circulating EVs after stroke that might reflect a disease specific process [46], even though the presence of comorbidities and their influence should be further investigated. EVs in stroke patients seem to mirror the pro-inflammatory nature of the events occurring after stroke, and their specific release was proposed as a specific brain signal to the periphery aimed at signaling the brain injury [46]. Going deep into the source of blood EVs, 24 h after induced stroke-reperfusion a significant increase of astrocytic EVs was observed in a mouse model of IS [47]. Numerous studies have focused on the crucial role of microglia as resident brain immune cells in the inflammatory process and in the reduction of stroke damage, and specifically the role of microglia-derived EVs in post-stroke phase. It must be noted that microglia activity is not limited to the secretion of inflammatory mediators, but microglia-derived EVs were also proved to act on oligodendrocyte functionality, favoring their maturation and thus the re-myelination process at the lesion site after stroke [48]. At the same time, in a model of in vitro ischemia, treatment of cortical neurons subjected to oxygen/glucose deprivation with oligodendrocyte-derived EVs reduced ischemic neuronal death by the EV-mediated transfer of superoxide dismutase and catalase, enzymes that are known to help cells to resist oxidative stress [49], another example of the complex interplay of brain cells and their EV signals. Collectively, EVs mainly protect the ischemic brain by mediating immune response, inhibiting brain cell apoptosis and inducing vascular remodeling and regeneration [50]. 

Finally, it was suggested that the prion protein PrP was enriched in EVs after stroke, with consequences in the regulation of vesicle uptake by recipient cells; the lack of PrP increases EV uptake by neurons, microglia, and astrocytes, suggesting a crucial role of PrP in the signaling mechanisms among brain cells after stroke [47]. 

## 6. Extracellular Vesicles as Stroke Biomarkers

A good biomarker candidate for the acute phase in stroke patients must be released in the hyper-acute phase and consistently over time, to be unique for stroke subtypes and easily measurable with simple devices. EVs represent a unique molecular window to the brain; once BBB becomes leaky or, even worse, there is BBB breakdown, the molecular cargo of EVs provides a plethora of accessible biomarkers on the brain health status and of the physiological and pathological processes occurring within the nervous tissue. The emerging role of EVs in cell–cell communications during the events that are triggered by the stroke events have raised interest in their use as accessible, measurable biomarkers. Circulating EVs that originated from brain cells of stroke patients at different pathological phases would be a dynamic and powerful tool for the screening, diagnosis, prognosis, and treatment efficiency monitoring for stroke. 

### 6.1. Extracellular Vesicles in Stroke Diagnosis

The EV analysis for stroke diagnosis must be rapid and simple. Classical EV isolation techniques are well-studied but time consuming, equipment-dependent, and not suitable for point of care setups. An optimal setup for this purpose must guarantee isolation and detection of EVs associated with stroke markers within minutes using whole blood. Multiplexed phenotyping of EV surface markers using antibodies would be a possible application for point-of-care tests, using a lab-on-a-chip microfluidic that combines separation, sorting, and detection with small sample volumes [51].

Among those studies aimed at the identification of stroke biomarkers, Simak et al. [52] found elevated expression of phosphatidylserine and CD105 and reduced expression of CD41a in endothelial EVs circulating in blood of acute stroke patients taken within 48 h from the event. Analyzing the circulating EVs from stroke patients, hyperexpression of Annexin-V was found in EVs from neural progenitor cells, platelets, endothelial cells, erythrocytes, and leucocytes [53]. Moreover, considering the concentration of vesicles instead of specific molecular markers, acute IS patients were found to have higher blood levels of EVs from activated platelets compared to patients with transient ischemic attack (TIA) [54]. Interestingly, as recently reviewed [8], several authors have reported the elevation of specific EV populations in stroke patients after 48 h from the event; nonetheless, the reasons for this increment are not fully understood and could depend on leakiness of the BBB, inflammatory response, or edema.

EV-associated biomarkers such as miRNA have attracted increasing attention as post-transcriptional regulators of gene expression and early biomarkers in many disorders, including stroke. For example, after isolation of blood EV, miR-134 [55], miR-9, and miR-124 [56] were found to be increased in IS patients in the acute phase and correlated with infarct volume and NIHSS scores. Additionally, miR-422a, miR-21-5p, and miR-30a-5p showed an initial peak in blood of IS patients followed by a decrease in the subacute phase [8]. The reported data about EV-associated miRNA in stroke suggest, up to now, the potentiality of such biomarkers to mirror the elapsed time from stroke, thanks to their fluctuations in blood [8], although limitations might rely in the variability of results related to isolation method used for EVs and blood collection timing. Reported studies often investigate single or few EV-associated mi-RNA at a time. However, to increase specificity and sensitivity of the analysis, the investigation of a wider panel of miRNA would be desirable, as suggested by Kalani and colleagues, who could differentiate HS and IS by means of a specific subset of EV-associated mi-RNAs [57]. Finally, the analysis of mRNA from CD8-positive EVs was proposed to diagnose acute IS by means of an innovative microfluidic device coupled with droplet digital PCR [58].

### 6.2. Extracellular Vesicles in the Prediction and Monitoring of Stroke Recovery 

The search for prognostic markers that can predict the recovery after stroke has gained considerable interest in recent years, especially in the rehabilitation medicine field. Indeed, the challenge of rehabilitation trials for stroke patients has progressively emerged; differences in timing (most rehabilitation is delivered within the first 30 days after stroke, yet less than 10% of motor rehabilitation trials are initiated during this time [59]), protocols, and limited sample sizes hamper the design of evidence-based rehabilitation protocols and the development of modelling studies. The use of a combination of biomarkers (clinical, instrumental, and biochemical) can provide clinically useful information when planning the personalized rehabilitation of a patient, and they could be used for patient selection and stratification in trials investigating rehabilitation interventions. Prediction tools that combine information in a systematic way could be used by clinicians to improve the accuracy of prognoses and personalize rehabilitation plans [60]. 

Starting from the pioneering work by Wagner [61], this concept was summarized in one word: **rehabilomics**. Rehabilomics refers to the “-omics”-based approach, i.e., in the use of molecular tools to understand the underlying mechanisms of current treatments and to systematically guide personalized treatment strategies in order to optimize rehabilitation outcomes and patient recovery. The introduction of rehabilomics for the personalized definition of the rehabilitation plan would speed up the achievement of specific goals, possibly bringing to a three-phase model the previously cited rehabilitation cycle [17] that included assessment, goal setting, intervention, and reassessment (Figure 2).

In the case of stroke, inflammatory markers related to the underlying mechanisms of pathogenesis and recovery have been investigated [11]. Among the emerging circulating markers [11], BDNF is certainly one of the most studied. Its low concentration in blood in the acute phase of IS is considered a negative prognosis factor; on the contrary, physical exercise can increase local levels of BDNF in the brain, leading to improvement in stroke recovery [62,63]. 

Trying to overcome the limitations of single molecule markers, EVs have started to emerge. Indeed, EV-associated molecules can rely on the possibility to perform a pre-isolation of brain-derived vesicles, or even a cell specific purification. In the search for a potential biomarker of recovery, the study of the role of EVs in stroke remodeling and response injury is emerging and could have a great clinical impact. For example, it was demonstrated that, 24 h after stroke, PrP expression in brain small EVs is increased, with functional consequences in intercellular communication after stroke [47].

Stroke long term and chronic EV biomarkers (released after 48 h from the acute event) are being investigated as well [8], because there is a clinical need for predictive biomarkers for stroke progression, follow-up, and treatment monitoring. 

Finally, it must be noted that EVs have also gained attention for their role in the communication between neural cells during tissue remodeling. Indeed, neuronal EVs have been suggested to transport proteins and miRNAs involved in synaptic plasticity, while microglia-derived EVs were proved to stimulate synaptic regeneration and remyelination thanks to surface molecules, such as lipids [43,64]. 

## 7. Extracellular Vesicles in Stroke Therapy

EVs have become an intense field of research, not only because they represent a novel form of cell-to-cell communication able to bridge wide distances, but also by their potential applicability as therapeutic tools. In the case of brain disorders, they are very attractive because of their intrinsic property to cross the BBB that can be enhanced thanks to specific modification able to target the brain tissue. 

Using surface functionalized EVs, EV-loaded curcumin (an anti-inflammatory compound) [65] and miR-210 [66] were delivered to the ischemic brain of mice and achieved significant treatment efficacy. The functionalization of the EV surface proposed by Tian and colleagues allowed for the improvement of the targeting ability of EVs after tail vein injection in a mouse model of cerebral ischemia, favoring also the uptake of curcumin-loaded EVs by neuron, microglia, and astrocytes, thus suppressing the pro-inflammatory cytokines and cellular apoptosis in the stroke lesion [65]. Similarly, promising results derived from the modification in the EV cargo for the specific delivery in miRNA, such as for example miR-124, which was suggested to promote cortical neural progenitors to obtain neuronal identity and also to protect against ischemic injury by robust cortical neurogenesis [67]. Engineered EVs were used also for the delivery of nerve growth factor (NGF) to the ischemic cortex of mouse brain; EVs containing mRNA and NGF proteins were injected through the tail vein 24 h post-ischemia and were proved to be uptaken by neurons, astrocytes, and microglia cells to reduce inflammation by reshaping microglia polarization and to promote cell survival [68].

In order to enhance the regeneration of the injured tissue with a complex mixture of bioactive molecules, mesenchymal stem/stromal cells (MSCs) have been proposed for tissue regeneration after stroke. However, since the first report of the use of MSC-derived EVs for regenerative purposes in a kidney failure model [69], vesicles have gained immediate interest. Actually, compared to their cellular counterparts, MSC-derived EVs maintain a positive effect on regeneration and immunomodulatory capacity, and they have considerable handling advantages, which can accelerate their clinical application in the so-called cell therapy without cells. Moreover, MSC-derived EVs overcome the risk of administer living, replicating, and difficult to control cells that challenges cell therapy in regenerative medicine [70].

MSC-released EVs may regulate neural survival, apoptosis, proliferation, and regeneration following brain damage and were proved to promote neural repair and functional recovery in animal models of ischemic stroke. Indeed, it was reported that intravenous delivery of EVs released by MSCs of bone-marrow origin improved axonal plasticity and neurite remodeling in the ischemic cortex in rats, possibly by the transfer of miRNA-133b to astrocytes and neurons [71]. Similarly, adipose tissue-derived-MSC released EVs can improve functional recovery and axonal sprouting after HS as well as oligodendrogenesis and white matter fiber repair when intravenously administered [72]. The pro-regenerative effect of MSC-derived EVs might also be related to their neuroprotective action demonstrated on cells undergoing glutamate-induced damage [73] as well as their ability to improve angiogenesis following cerebral ischemia [74]. Actually, it was demonstrated that intravenous administration of MSC-derived EVs increases the percentage of newly formed cells expressing endothelial markers (von Willebrand factor-positive cells) in the ischemic zone [74]. Compared with PBS-treated controls, EV treatment increased the recovery after stroke, measured as improvement in neurovascular plasticity in the stroke affected hemisphere, and promotion of neurological function recovery. 

Together with the pro-regenerative effects, MSC-derived EVs might favor the recovery after stroke thanks to their immunomodulatory properties. EVs from bone-marrow-derived MSC were actually proved to influence and modulate the immune reactions in the injured brain following focal cerebral ischemia, thus proving an appropriate external milieu for successful brain remodeling [75]. 

It is also important to mention that various approaches are currently being employed to drive the MSC secretome toward a more anti-inflammatory and regenerative phenotype [76,77], and multiple stem cell sources have been proposed [78,79] as well as a 3D-culture system for large scale expansion of cells to increase the EV yield. Among the EV sources, evidence has demonstrated the ability of EVs from cerebral endothelial cells to enhance axonal growth by the upregulation of miRNAs associated with targeted reduction of axonal inhibitory proteins in recipient neurons [80]. EVs derived from neural progenitor cells (NPC) were also tested in vitro on cerebral organoids exposed to oxygen–glucose deprivation and in vivo in mice following experimental ischemia, demonstrating enhanced neurological recovery and neuro-regeneration for as long as 3 months [81]. However, the therapeutic impact of such NPC-EVs was found to be similar to that of MSC-EVs. Still, only a few clinical studies on the effects of EV therapy have been reported in humans, and, despite the reported potential advantages compared to the cellular counterparts, clinical evaluation of EV therapeutics is still at an early stage [82,83]. 

In a recent study, the pro-regenerative effect of microglia-derived EVs was also proposed in a mouse model of IS, with specific positive effects on oligodendrocyte progenitor cell proliferation and maturation [48]. In particular, the beneficial action was proved in the early post-ischemic phase, showing that EVs collected from microglia cultured with a pro-regenerative stimulus are able to stimulate myelin repair in neurological recovery [48].

It must be noted that several aspects of EV therapeutic application are still to be elucidated, not only concerning the EV source but also the dosing and timing of treatment and in combination with standard pharmacological therapy, such as thrombolysis with tissue plasminogen activator for acute IS, and with neurorehabilitation [84]. 

Finally, in the perspective of the regenerative rehabilitation approach, it must be mentioned that in the therapeutic approach to stroke, EVs have attracted interest also for their role in the functional recovery mediated by motor rehabilitation. Physical exercise induces the release of EVs into circulation, with proteins demonstrated to circulate in/on vesicles during exercise, allowing the crosstalk between muscles and brain (Figure 1c) [85]. In addition to endogenous repair processes and exercise induced repair, it has been reported that exercise is highly beneficial for recovery from stroke after transplantation of MSCs and that both motor rehabilitation and stem-cell therapy worked synergistically to mitigate apoptosis and favor recovery [86]. Starting from these premises, the regenerative rehabilitation strategy described above might be considered an innovative approach to enhance the pro-regenerative effects of MSC-derived EVs (or other EV populations) after stroke, although no studies have been reported yet. 

## 8. Conclusions

In recent years, new solutions have been proposed for stroke therapy and rehabilitation intervention after stroke. Among them, EVs have gained interest for both their possible use as biomarkers and as therapeutics. Indeed, their involvement in the main processes occurring after stroke such as the inflammatory cascade, spontaneous regeneration, and remodeling have emerged, significantly inspiring researchers all over the world. 

The literature review reported in the present paper demonstrates the considerable potential of EVs as biomarkers in the acute phase after stroke, but, above all, in the prognosis and prediction of the outcome of therapeutic and rehabilitation intervention. The crucial role of a personalized rehabilitation program for optimal patient recovery has highlighted the need for rehabilitation markers. It must be noted that in the search for EV application in rehabilomics, technological advancements are needed to ameliorate the ability to detect variations in the EV cargoes in accessible biofluids as well as the standardization of the operating procedures that should fit a clinical setting and guarantee a fast execution. At the same time, we would like to underline the need for proper design of rehabilitation trials that could shed light on the actual strength of the recently proposed markers, including the EV-associated cargoes such as miRNAs, neurotrophic factors, and others.

Concomitantly, the regenerative rehabilitation field is still in its infancy, but we foresee that promising advancements might result from the combination of EV-based therapies and advanced rehabilitation trials. Indeed, the results of EV application in regeneration and rehabilitation recovery after stroke would have a great impact on the aging society, both for the resulting benefits for the patients’ quality of life and for the economically-positive effects on the health care system.

## Data Availability

Not applicable.
